# Data on genetic potentiality of folk rice (*Oryza sativa* L.) genotypes from Koraput, India in reference to drought tolerance traits

**DOI:** 10.1016/j.dib.2019.104363

**Published:** 2019-08-12

**Authors:** Debabrata Panda, Swati S. Mishra, Sangram K. Mohanty, Prafulla K. Behera, Sangram K. Lenka

**Affiliations:** a Department of Biodiversity and Conservation of Natural Resources, Central University of Orissa, Koraput, 764 021, Odisha, India; b National Rice Research Institute (ICAR), Cuttack, 753 006, Odisha, India; c TERI-Deakin NanoBiotechnology Centre, The Energy and Resources Institute, Gurugram, Haryana, 122 001, India

**Keywords:** Drought tolerance, Indigenous rice, Simple sequence repeat, Microsatellite panel

## Abstract

Precise physiological and molecular marker-based assessment provides information about the extent of genetic diversity, which helps for effective breeding programmes. We have conducted detailed physiological and molecular marker-based assessment of selected eight indigenous rice landraces from Koraput, India along with tolerant (N22) and susceptible (IR64) check varieties under control and simulated drought stress using polyethylene glycol (PEG) 6000. After exposure to different levels of drought stress, relative germination performance (RGP), seedling vigour index (SVI) and relative growth index (RGI) were significantly declined in all the rice landraces compared to the control plants and significant varietal differences were observed. Genetic relationship among the studied rice landraces was assessed with 24 previously reported drought tolerance linked Simple Sequence Repeat (SSR) markers. A total of 53 alleles were detected at the loci of the 24 markers across the 10 rice accessions**.** The Nei's gene diversity (*He*) and the polymorphism information content (*PIC*) ranged from 0 to 0.665 and 0 to 0.687, respectively. Six SSR loci, RM276, RM411, RM3, RM263, RM216 and RM28199, provided the highest *PIC* values and are potential for exploring the genetic diversity of studied rice lines for drought tolerance. Four rice genotypes (Butkichudi, Haldichudi, Machakanta and Kalajeera) showed the highest genetic distance with tolerant check variety (N22) and can be considered as valuable genetic resources for drought breeding program.

**Table:** Specifications table

Subject area	Biology
More specific subject area	Plant Physiology and Molecular Biology
Type of data	Table, figures
How data was acquired	The seedling vigour characteristics of 15-days-old seedlings were measured by taking root and shoot length, fresh and dry weight under drought as well as at control conditions. Molecular genotyping was carried out by taking 10 rice genotypes with 24 SSR markers linked with drought tolerance QTL. Total genomic DNA was extracted and purified from the young leaves and PCR reaction was performed in thermal cycler (BioRad, USA).
Data format	Raw and analyzed data
Experimental factors	After sowing seeds, immediately the drought stress was simulated with variations in osmotic potential by application of different concentrations (19.6%, 29.6% and 36.0%) of polyethylene glycol (PEG) that produced −0.5, −1.0 and −1.5 MPa water potential, respectively for 15 days.
Experimental features	Determination of early growth performances, Genotyping with drought tolerance linked rice microsatellite loci, Isolation of genomic DNA, PCR amplification, polymorphism screening and analysis of amplified products.
Data source location	Laboratory of Central University of Orissa, Koraput (82˚44̕ʹ54ʹʹ E to 18˚46̕ʹ47ʹʹ N), India
Data accessibility	Data is available with this article
Related research article	Mishra and Panda [1] Leaf traits and antioxidant defense for drought tolerance during early growth stage in some popular traditional rice landraces from Koraput, India. Rice Sci. 24 (2017) 207–217.

**Table:** 

**Value of the data** • The first open-access visual feature data set that describes genetic potentiality of folk rice (*Oryza sativa* L.) genotypes from Koraput for drought tolerance traits. • Our data provides information about the description of variety, tables and graphs for physiological and molecular analysis and also microsatellite panel along with the genetic relationship. Hence, it gives a holistic and clear view of genetic potentiality of the studied genotypes for drought tolerance. • The data presented can be a benchmark to conserve the existing rice gene pool as well as popularization of the rice genotypes for future breeding programs.

## 1 Data

**Fig. 1:**
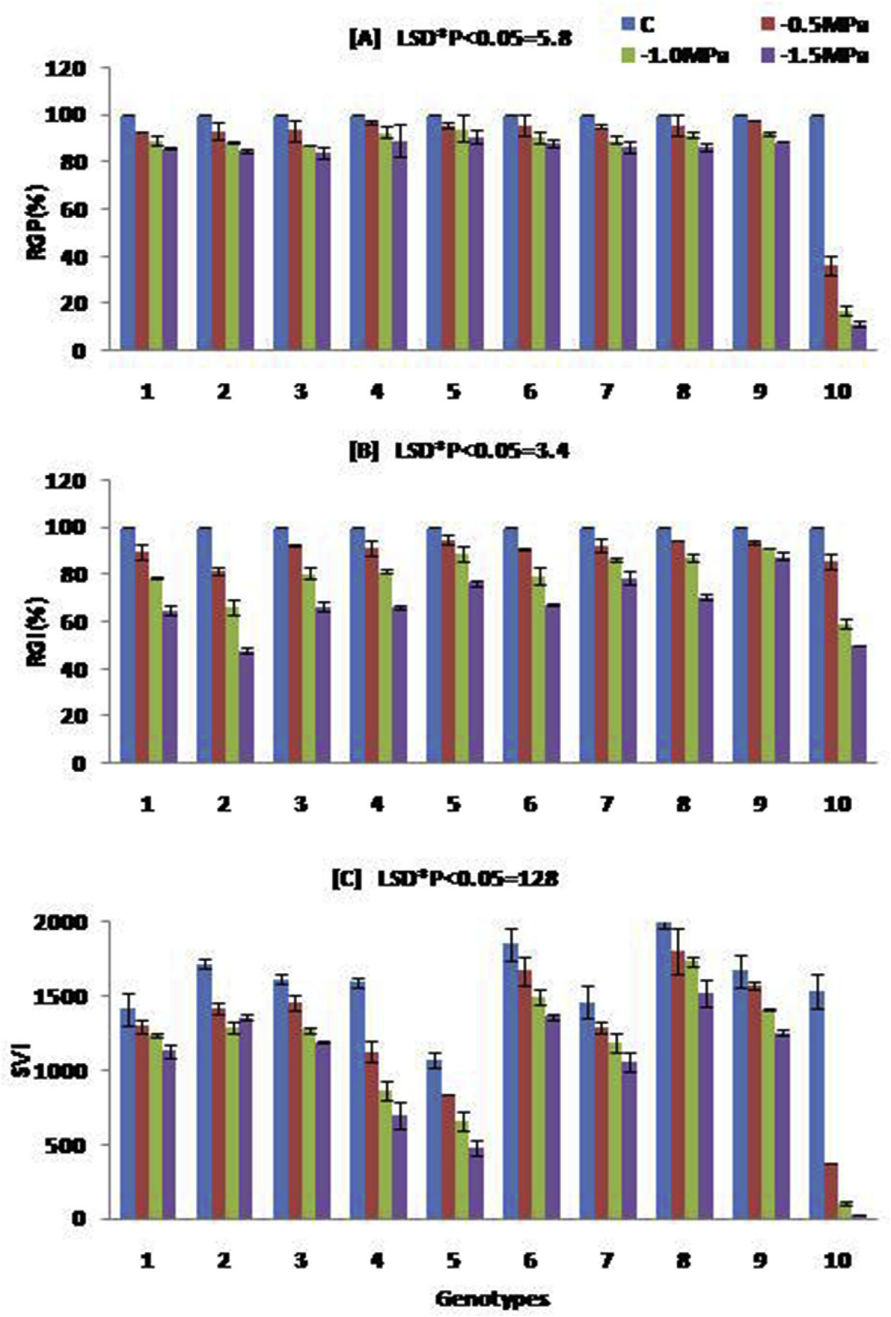
Variations of relative germination performance (RGP), relative growth index (RGI) and seedling vigour index (SVI) of studied rice genotypes in different concentration of PEG induced drought stress. Data are the mean of three replications (n = 3) with vertical bar represents standard deviation. The treatment C: control and −0.5 MPa, −1.0 MPa and −1.5 MPa are different levels of drought. LSD: least significance difference. Genotypes 1: Dangarabayagundar; 2: Machhakanta; 3:Kalajeera; 4:Butukichud; 5:Bhatachudi; 6:Haladichudi; 7:Pandakagura; 8:Mugudi; 9:N 22; 10:IR 64.

**Fig. 2:**
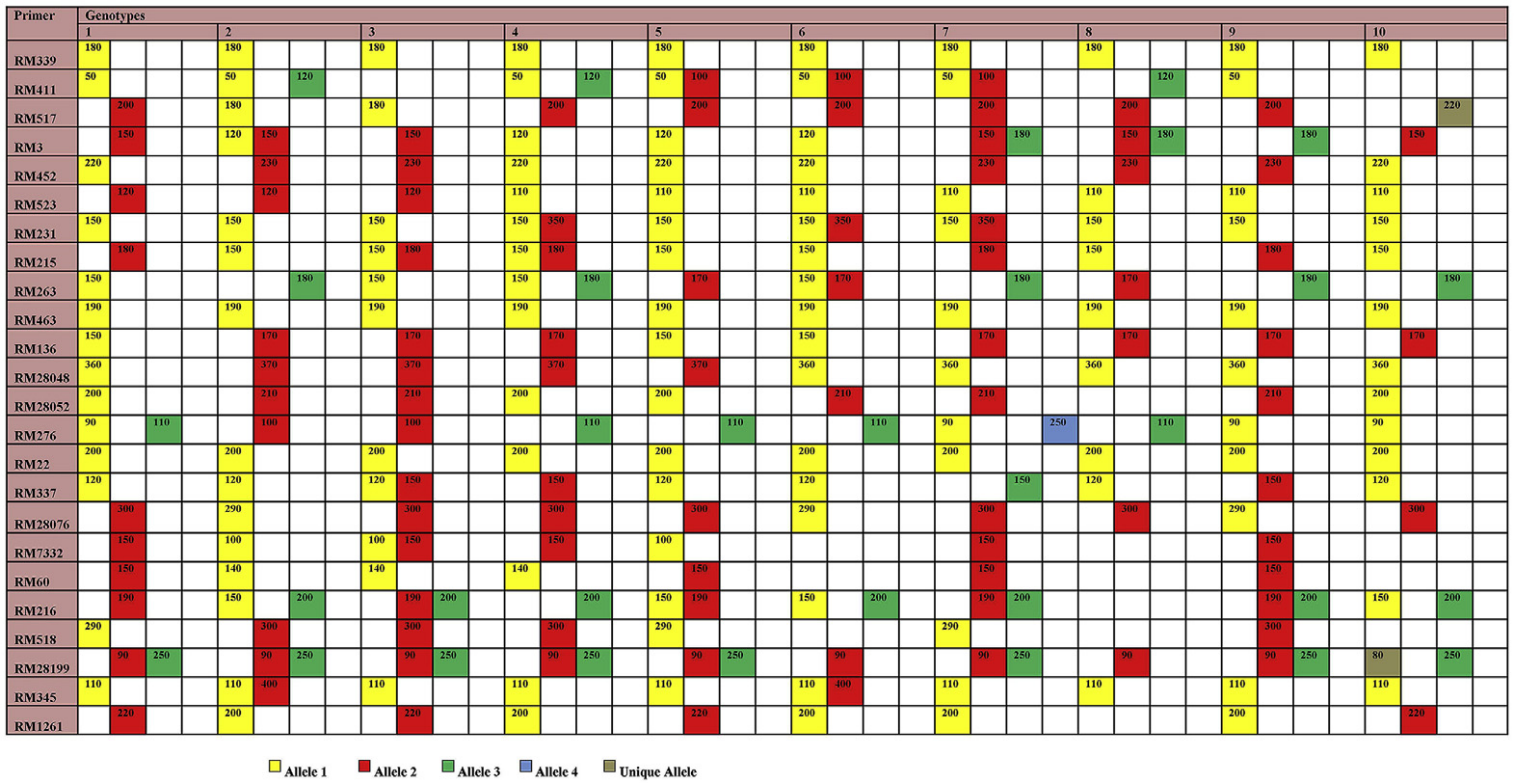
Microsatellite panel of studied SSR primers in different genotypes. Genotypes 1: Dangarabayagundar; 2: Machhakanta; 3:Kalajeera; 4:Butukichudi; 5:Bhatachudi; 6:Haladichudi; 7:Pandakagura; 8:Mugudi; 9:N 22; 10:IR 64.

**Fig. 3:**
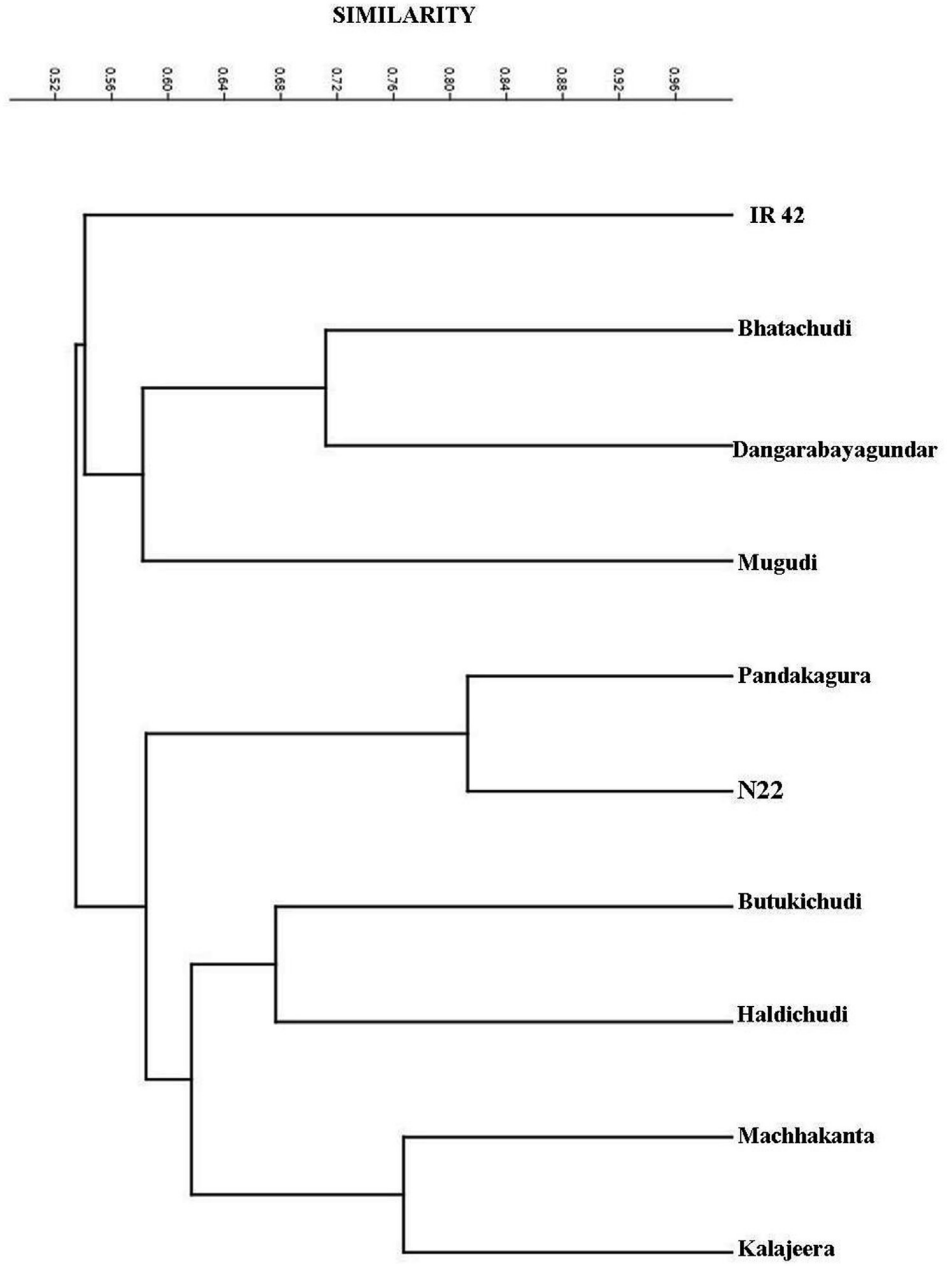
Dendrogram showing the percentage of similarity between the rice genotypes based on SSR amplified products.

The dataset contains tables, graphs and images derived from the analysis of the raw data obtained from the various growth and genetic diversity parameters of the folk rice varieties from Koraput, India under control and drought condition. Details of genotypes with their origin, ecotype and special characters were presented in Table 1. Variations of relative germination performance (RGP), relative growth index (RGI) and seedling vigour index (SVI) of studied rice genotypes in different concentration of PEG induced drought stress was shown in Fig. 1. Analysis of variance (ANOVA) of studied parameters in rice seedlings grown under different levels drought stress was presented in Table 2. Genotyping of the studied genotypes was carried out by taking 24 reported simple sequence repeat (SSR) markers linked to different drought tolerance QTL [2] and details of SSR markers are presented in Table 3. Different alleles, in form of variation in molecular weight of each amplified products for each SSR marker against studied ten genotypes are given in a Microsatellite Panel (Fig. 2). The markers amplified a total of 53 alleles with an average of 2.2 per locus. Genetic diversity parameters such as number of alleles, number of effective alleles, expected homozygosity, expected heterozygosity, Nei's genetic diversity, Shannon's information index and polymorphism information content was presented in Table 4. The pair-wise genetic similarity calculated for all the studied genotypes with 24 SSR markers ranged from 0.431 to 0.813 (Table 5). Cluster analysis based on the Bray-Curtis paired linkage revealed the percent of similarity in SSR marker data among studied rice genotypes were presented in Fig. 3.

**Table 1:** Details of genotypes with their origin, ecotype and special characters.

Variety No	Variety	Origin	Ecotype	Characters
1	Dangarabayagundar	Landraces of Koraput	Up land	Short Duration of maturity (103 days), medium and bold grain, white grain, coarse white rice, drought escaping.
2	Machhakanta	Landraces of Koraput	Low land	Long duration of maturity (135–145 days), slender grain, popular variety, drought tolerant.
3	Kalajeera	Landraces of Koraput	Low land	Long duration of maturity (140–150 days), aromatic, small oval grain with black husk color
4	Butukichudi	Landraces of Koraput	Low land	Long duration of maturity (135–140 days), brown grain, white coarse rice, strong straw and drought tolerant.
5	Bhatachudi	Landraces of Koraput	Medium land	Medium duration of maturity (130 days), yellow grain, white coarse rice, non-lodging, white seed coat color, drought tolerant.
6	Haladichudi	Landraces of Koraput	Medium land	Medium duration of maturity (125–135 days), medium slender grain, deep yellow husk color, popular variety, drought tolerant.
7	Pandakagura	Landraces of Koraput	Up land	Long duration of maturity (145–150 days), drought tolerant, white grain, coarse white rice.
8	Mugudi	Landraces of Koraput	Low land	Medium duration of maturity (130–135 days), drought tolerant, strong straw, bold kernel.
9	N 22	Eastern India	Up land	Short duration of maturity (80–95 days), deep-rooted, drought and heat tolerant *aus* rice variety.
10	IR 64	IRRI, Philippines	Low land	Short duration of maturity (115 days), high yielding hybrid variety, long slender grain, rainfed lowland area, susceptible to drought stress.

**Table 2:** Sum square is the absolute value and percentage of total (in bracket) of main effect resulting from analysis of variance (ANOVA) of studied parameters in rice seedlings grown under different levels drought stress. df, Degrees of freedom; Total df = 49; The *P* of overall ANOVA for variety, treatment and variety × treatment interaction for each parameters *P < 0.05,**P < 0.01.

Parameters	Source of Variation		
	Variety (df = 9)	Treatment (df = 3)	Variety × Treatment (df = 37)
RGP	19571** (62)	4760** (15)	6843** (21)
RGI	2993** (18)	11611** (70)	1834** (11)
SVI	1046610** (64)	384915** (23)	174011** (10)

RGP: relative germination performance; RGI: relative growth Index; SVI: seedling vigour index.

**Table 3:** Details of SSR markers used in this study.

Sl. No.	Primer		Repeat motive	Forward primer	Reverse	Chromosome No.
1	RM339		(CTT)8CCT (CTT)5	GTAATCGATGCTGTGGGAAG	GAGTCATGTGATAGCCGATATG	8
2	RM411		(GTT)7	ACACCAACTCTTGCCTGCAT	TGAAGCAAAAACATGGCTAGG	3
3	RM517		(CT)15	GGCTTACTGGCTTCGATTTG	CGTCTCCTTTGGTTAGTGCC	3
4	RM3		(GA)2GG (GA)25	ACACTGTAGCGGCCACTG	CCTCCACTGCTCCACATCTT	6
5	RM452		(GTC)9	CTGATCGAGAGCGTTAAGGG	GGGATCAAACCACGTTTCTG	2
6	RM523		(TC)14	AAGGCATTGCAGCTAGAAGC	GCACTTGGGAGGTTTGCTAG	3
7	RM231		(CT)16	CCAGATTATTTCCTGAGGTC	CACTTGCATAGTTCTGCATTG	3
8	RM215		(CT)16	CAAAATGGAGCAGCAAGAGC	TGAGCACCTCCTTCTCTGTAG	9
9	RM263		(CT)34	CCCAGGCTAGCTCATGAACC	GCTACGTTTGAGCTACCACG	2
10	RM463		(TTAT)5	TTCCCCTCCTTTTATGGTGC	TGTTCTCCTCAGTCACTGCG	12
11	RM136		(AGG)7	GAGAGCTCAGCTGCTGCCTCTAGC	GAGGAGCGCCACGGTGTACGCC	6
12	RM28048		(CGC)8	TTCAGCCGATCCATTCAATTCC	GCTATTGGCCGGAAAGTAGTTAGC	12
13	RM28052		(TA)26	ACTAAAGATCTTCGAGCTGACC	GCTACATGGAGTATGGGTTCC	12
14	RM276		(AG)8A3 (GA)33	CTCAACGTTGACACCTCGTG	TCCTCCATCGAGCAGTATCA	6
15	RM22		(GA)22	GGTTTGGGAGCCCATAATCT	CTGGGCTTCTTTCACTCGTC	3
16	RM337		(CTT)4-19-(CTT)8	GTAGGAAAGGAAGGGCAGAG	CGATAGATAGCTAGATGTGGCC	8
17	RM28076		(CT)13	GGGACTTGGGACCAGTTTATGG	TCAGGTCTGTTGGATTCCATGC	12
18	RM7332		(ACAT)11	ACACTGTACACCACACTTCAGC	CAGGGAAATGACACTGTCCC	3
19		RM60	(AATT)5AATCT (AATT)	AGTCCCATGTTCCACTTCCG	ATGGCTACTGCCTGTACTAC	3
20	RM216		(CT)18	GCATGGCCGATGGTAAAG	TGTATAAAACCACACGGCCA	10
21	RM518		(TC)15	CTCTTCACTCACTCACCATGG	ATCCATCTGGAGCAAGCAAC	4
22	RM28199		(ATAG)5	CGGCTTAGGGAGCGTCTGTAGG	GCATGCTAGTATGGCCACCATATTCC	12
23	RM345		(CTT)9	ATTGGTAGCTCAATGCAAGC	GTGCAACAACCCCACATG	6
24	RM1261		(AG)16	GTCCATGCCCAAGACACAAC	GTTACATCATGGGTGACCCC	12

**Table 4:** Genetic diversity parameters calculated on SSR data. *Na*: number of alleles; *Ne*: number of effective alleles; *Ho*: expected homozygosity; *H*e: expected heterozygosity/Nei's genetic diversity; *I*: Shannon's information index; PIC: polymorphism information content.

Locus	*Na*	*Ne*	*Ho*	*He*	*I*	PIC
RM339	1	1.000	1.000	0.000	0.000	0
RM411	3	2.415	0.375	0.586	0.984	0.603
RM517	3	1.852	0.516	0.460	0.802	0.460
RM3	3	2.740	0.332	0.635	1.049	0.639
RM452	2	2.000	0.474	0.500	0.693	0.500
RM523	2	1.724	0.558	0.420	0.611	0.420
RM231	2	1.342	0.732	0.255	0.423	0.355
RM215	2	1.923	0.495	0.480	0.673	0.486
RM263	3	2.817	0.321	0.645	1.067	0.661
RM463	1	1.000	1.000	0.000	0.000	0
RM136	2	1.724	0.558	0.420	0.611	0.420
RM28048	2	1.923	0.495	0.480	0.673	0.480
RM28052	2	1.976	0.477	0.494	0.687	0.493
RM276	4	2.985	0.300	0.665	1.192	0.687
RM22	1	1.000	1.000	0.000	0.000	0
RM337	2	1.835	0.521	0.455	0.647	0.462
RM28076	2	1.724	0.558	0.420	0.611	0.420
RM7332	2	1.849	0.506	0.459	0.652	0.468
RM60	2	1.960	0.473	0.490	0.683	0.489
RM216	3	2.946	0.301	0.661	1.089	0.648
RM518	2	1.960	0.473	0.490	0.683	0.489
RM28199	3	2.151	0.437	0.535	0.845	0.549
RM345	2	1.220	0.811	0.180	0.325	0.277
RM1261	2	1.976	0.477	0.494	0.687	0.493
**Mean**	**2.208**	**1.918**	**0.549**	**0.426**	**0.654**	**0.437**
**St. Dev**	**0.721**	**0.570**	**0.210**	**0.199**	**0.325**	**0.194**

**Table 5:** Similarity matrix of the studied rice genotypes on the basis of Bray Curtis similarity index. Genotypes 1: Dangarabayagundar; 2: Machhakanta; 3:Kalajeera; 4:Butukichudi; 5:Bhatachudi; 6:Haladichudi; 7:Pandakagura; 8:Mugudi; 9:N 22; 10:IR 64.

Genotypes	1	2	3	4	5	6	7	8	9	10
1	1.000									
2	0.431	1.000								
3	0.576	0.767	1.000							
4	0.563	0.648	0.667	1.000						
5	0.712	0.485	0.478	0.615	1.000					
6	0.516	0.638	0.514	0.676	0.635	1.000				
7	0.615	0.583	0.630	0.704	0.545	0.609	1.000			
8	0.549	0.483	0.475	0.561	0.615	0.545	0.586	1.000		
9	0.561	0.531	0.523	0.603	0.483	0.492	0.813	0.560	1.000	
10	0.545	0.516	0.540	0.525	0.536	0.508	0.548	0.542	0.481	1.000

## 2 Experimental design, materials, and methods

### 2.1 Plant materials and growth conditions

The experiment was conducted by taking eight folk rice genotypes from Koraput, India along with N22 (drought-tolerant improved rice variety) and IR64 (drought-susceptible irrigated variety) as check varieties. The details of the rice landraces used in this study are presented in Table 1. Uniform sized seeds of each variety were selected, surface sterilized and kept for germination. The seeds were placed in sterilized petriplates over saturated tissue paper and transferred to an incubator with a 12-h light/12-h dark photoperiod with daily maximum photosynthetic photon flux density (PPFD) about 380 ± 40 μ mol m^−2^ s^−1^ at 25 °C in the laboratory. After sowing seeds immediately the drought stress was simulated with variations in osmotic potential by application of different concentrations (19.6%, 29.6% and 36.0%) of polyethylene glycol (PEG) that produced −0.5, −1.0 and −1.5 MPa water potential, respectively for 15 days. A control set was also run along with the treatment without application of PEG.

### 2.2 Determination of early growth performances

The seed germination rate was recorded 9 days after sowing. The seedling vigour characteristics of 15-days-old seedlings were measured by taking root and shoot length, fresh and dry weight of five different plants in each replication under drought as well as at control conditions. For analysing early growth performances, relative germination performance (RGP), relative growth index (RGI), and seedling vigour index (SVI) were calculated according to Rubio-Casal et al. [3] and Bhattacharjee [4] as follows.

RGI = (average dry mass of five treated seedling/average dry mass of five control seedlings) × 100.

RGP = (percentage of germination under treatment/percentage of germination under control) × 100.

SVI = (mean shoot length +mean root length) × percentage of final germination.

### 2.3 Genotyping with drought tolerance linked rice microsatellite loci

Genotyping with drought tolerance linked rice microsatellite loci was done by taking 24 reported simple sequence repeat (SSR) markers linked to different drought tolerance QTL. Total genomic DNA was extracted and purified from the young leaves by a modified CTAB (cetyl-trimethylammonium bromide) method described by Murray and Thompson [5]. The PCR amplification was performed in thermal cycler (BioRad, USA) by taking 20 μl volumes mixed with 2 μl of genomic DNA (25 ng ml^−1^), 1.5 μl of MgCl_2_ (25 mM L^−1^), 0.3 μl of dNTP mixtures (10 mM L^−1^), 2 μl of 10 PCR buffer, 2 μl of SSR primer (2 mM L^−1^), 0.2 μl of Taq polymerase (10 U ml^−1^) and 12 μl of ddH_2_O, following the method of Panaud et al. [6]. The PCR amplification was an initial denaturation at 94 °C for 5 min followed by 35 cycles of denaturation at 94 °C for 30 sec, annealing (depending on TM value of primer) at 50–60 °C for 45 sec, extension at 72 °C for 1 min and a final extension of 7 min at 72 °C. The amplified products were resolved through 2.5% ethidium bromide stained (1 μg ml^−1^) agarose gel and documented using a gel documentation system (BioRad, USA). The different allelic forms (variation in molecular weight of the amplicons) of individual SSR loci were scored as 1 or 0 based on their presence or absence, respectively across the studied rice genotypes. A proximity matrix was constructed from the 1/0 matrix using PAST-3 (Palaeontological Statistics) software to construct a dendrogram using average linkage among the studied genotypes. Marker based population genetics study was performed with calculation of polymorphic information content (*PIC*), effective number of alleles (*Ne*), Shannon's Information index (*I*), and Nei's heterozygosity (*He*) was performed using genetic diversity analysis software POPGENE 1.31 [7].

### 2.4 Statistical analysis

Growth parameters were analyzed by two-way analysis of variance (ANOVA) with the variety and different treatment levels by using CROPSTAT (International Rice Research Institute, Philippines) software.
