# Supplementary material for: Data on genetic potentiality of folk rice (Oryza sativa L.) genotypes from Koraput, India in reference to drought tolerance traits
Source: Data Brief. 2019 Aug 12;25:104363. doi: 10.1016/j.dib.2019.104363 (PMC6715829; doi:10.1016/j.dib.2019.104363)
Supplement: Multimedia component 1 [file mmc1.docx]

**Table S1** Raw data of relative germination performance (RGP), relative growth index (RGI) and seedling vigour index (SVI) of studied rice genotypes in different concentration of PEG induced drought stress. The treatment C: control and -0.5 MPa, -1.0 MPa and -1.5 MPa are different levels of drought.

| **Variety** | **Treatment** | **Replication** | **Relative germination performance** | **Relative growth index (RGI)** | **Seedling vigour index** |
| --- | --- | --- | --- | --- | --- |
| Dangarabayagundar | C | 1 | 100.00 | 100.00 | 1493.80 |
|  |  | 2 | 100.00 | 100.00 | 1339.50 |
|  |  | 3 | 100.00 | 100.00 | 1416.65 |
|  | -0.5 MPa | 1 | 92.78 | 87.57 | 1332.00 |
|  |  | 2 | 92.63 | 92.08 | 1267.20 |
|  |  | 3 | 92.71 | 89.82 | 1299.60 |
|  | -1.0 MPa | 1 | 87.63 | 78.70 | 1224.00 |
|  |  | 2 | 90.53 | 79.38 | 1247.00 |
|  |  | 3 | 89.08 | 79.04 | 1235.50 |
|  | -1.5 MPa | 1 | 85.57 | 63.85 | 1162.00 |
|  |  | 2 | 86.32 | 66.24 | 1098.80 |
|  |  | 3 | 85.94 | 65.04 | 1130.40 |
| Machhakanta | C | 1 | 100.00 | 100.00 | 1699.20 |
|  |  | 2 | 100.00 | 100.00 | 1739.00 |
|  |  | 3 | 100.00 | 100.00 | 1719.10 |
|  | -0.5 MPa | 1 | 90.63 | 80.60 | 1392.00 |
|  |  | 2 | 95.74 | 83.04 | 1449.00 |
|  |  | 3 | 93.18 | 81.82 | 1420.50 |
|  | -1.0 MPa | 1 | 88.54 | 64.03 | 1317.50 |
|  |  | 2 | 88.30 | 68.24 | 1261.60 |
|  |  | 3 | 88.42 | 66.13 | 1289.55 |
|  | -1.5 MPa | 1 | 84.38 | 47.11 | 1344.60 |
|  |  | 2 | 85.11 | 48.65 | 1368.00 |
|  |  | 3 | 84.74 | 47.88 | 1356.30 |
| Kalajeera | C | 1 | 100.00 | 100.00 | 1590.30 |
|  |  | 2 | 100.00 | 100.00 | 1634.00 |
|  |  | 3 | 100.00 | 100.00 | 1612.15 |
|  | -0.5 MPa | 1 | 96.77 | 92.55 | 1422.00 |
|  |  | 2 | 90.53 | 92.21 | 1487.80 |
|  |  | 3 | 93.65 | 92.38 | 1454.90 |
|  | -1.0 MPa | 1 | 87.10 | 82.17 | 1287.90 |
|  |  | 2 | 87.37 | 78.86 | 1253.30 |
|  |  | 3 | 87.23 | 80.51 | 1270.60 |
|  | -1.5 MPa | 1 | 86.02 | 68.21 | 1184.00 |
|  |  | 2 | 82.11 | 65.45 | 1193.40 |
|  |  | 3 | 84.06 | 66.83 | 1188.70 |
| Butukichudi | C | 1 | 100.00 | 100.00 | 1566.40 |
|  |  | 2 | 100.00 | 100.00 | 1618.20 |
|  |  | 3 | 100.00 | 100.00 | 1592.30 |
|  | -0.5 MPa | 1 | 96.59 | 93.88 | 1079.50 |
|  |  | 2 | 97.85 | 88.94 | 1173.90 |
|  |  | 3 | 97.22 | 91.41 | 1126.70 |
|  | -1.0 MPa | 1 | 90.91 | 82.14 | 816.00 |
|  |  | 2 | 94.62 | 80.71 | 915.20 |
|  |  | 3 | 92.77 | 81.43 | 865.60 |
|  | -1.5 MPa | 1 | 95.45 | 66.84 | 764.40 |
|  |  | 2 | 82.80 | 65.74 | 639.10 |
|  |  | 3 | 89.13 | 66.29 | 701.75 |
| Bhatachudi | C | 1 | 100.00 | 100.00 | 1105.80 |
|  |  | 2 | 100.00 | 100.00 | 1035.00 |
|  |  | 3 | 100.00 | 100.00 | 1070.40 |
|  | -0.5 MPa | 1 | 94.85 | 93.57 | 837.20 |
|  |  | 2 | 96.67 | 96.24 | 843.90 |
|  |  | 3 | 95.76 | 94.91 | 840.55 |
|  | -1.0 MPa | 1 | 87.63 | 86.62 | 612.00 |
|  |  | 2 | 101.11 | 91.46 | 709.80 |
|  |  | 3 | 94.37 | 89.04 | 660.90 |
|  | -1.5 MPa | 1 | 92.78 | 75.89 | 513.00 |
|  |  | 2 | 88.89 | 77.59 | 440.00 |
|  |  | 3 | 90.84 | 76.74 | 476.50 |
| Haladichudi | C | 1 | 100.00 | 100.00 | 1930.60 |
|  |  | 2 | 100.00 | 100.00 | 1776.30 |
|  |  | 3 | 100.00 | 100.00 | 1853.45 |
|  | -0.5 MPa | 1 | 92.86 | 91.05 | 1601.60 |
|  |  | 2 | 98.92 | 90.56 | 1738.80 |
|  |  | 3 | 95.89 | 90.81 | 1670.20 |
|  | -1.0 MPa | 1 | 88.78 | 76.66 | 1461.60 |
|  |  | 2 | 92.47 | 81.95 | 1530.80 |
|  |  | 3 | 90.62 | 79.31 | 1496.20 |
|  | -1.5 MPa | 1 | 86.73 | 67.71 | 1351.50 |
|  |  | 2 | 89.25 | 67.44 | 1377.80 |
|  |  | 3 | 87.99 | 67.57 | 1364.65 |
| Pandakagura | C | 1 | 100.00 | 100.00 | 1536.00 |
|  |  | 2 | 100.00 | 100.00 | 1381.80 |
|  |  | 3 | 100.00 | 100.00 | 1458.90 |
|  | -0.5 MPa | 1 | 94.79 | 90.71 | 1319.50 |
|  |  | 2 | 95.74 | 94.31 | 1260.00 |
|  |  | 3 | 95.27 | 92.51 | 1289.75 |
|  | -1.0 MPa | 1 | 90.63 | 86.17 | 1235.40 |
|  |  | 2 | 88.30 | 87.39 | 1145.40 |
|  |  | 3 | 89.46 | 86.78 | 1190.40 |
|  | -1.5 MPa | 1 | 88.54 | 76.46 | 1105.00 |
|  |  | 2 | 85.11 | 80.52 | 1016.00 |
|  |  | 3 | 86.82 | 78.49 | 1060.50 |
| Mugudi | C | 1 | 100.00 | 100.00 | 1976.00 |
|  |  | 2 | 100.00 | 100.00 | 2038.40 |
|  |  | 3 | 100.00 | 100.00 | 2007.20 |
|  | -0.5 MPa | 1 | 92.63 | 94.79 | 1698.40 |
|  |  | 2 | 98.90 | 94.88 | 1908.00 |
|  |  | 3 | 95.77 | 94.83 | 1803.20 |
|  | -1.0 MPa | 1 | 88.42 | 88.48 | 1713.60 |
|  |  | 2 | 94.51 | 86.34 | 1754.40 |
|  |  | 3 | 91.46 | 87.41 | 1734.00 |
|  | -1.5 MPa | 1 | 85.26 | 71.35 | 1579.50 |
|  |  | 2 | 87.91 | 69.70 | 1456.00 |
|  |  | 3 | 86.59 | 70.52 | 1517.75 |
| N 22 | C | 1 | 100.00 | 100.00 | 1590.80 |
|  |  | 2 | 100.00 | 100.00 | 1747.20 |
|  |  | 3 | 100.00 | 100.00 | 1669.00 |
|  | -0.5 MPa | 1 | 97.94 | 93.27 | 1586.50 |
|  |  | 2 | 97.92 | 94.31 | 1551.00 |
|  |  | 3 | 97.93 | 93.79 | 1568.75 |
|  | -1.0 MPa | 1 | 92.78 | 91.13 | 1413.00 |
|  |  | 2 | 91.67 | 91.54 | 1408.00 |
|  |  | 3 | 92.23 | 91.34 | 1410.50 |
|  | -1.5 MPa | 1 | 88.66 | 86.75 | 1247.00 |
|  |  | 2 | 88.54 | 89.04 | 1266.50 |
|  |  | 3 | 88.60 | 87.89 | 1256.75 |
| IR 64 | C | 1 | 100.00 | 100.00 | 1612.80 |
|  |  | 2 | 100.00 | 100.00 | 1447.60 |
|  |  | 3 | 100.00 | 100.00 | 1530.20 |
|  | -0.5 MPa | 1 | 33.33 | 87.93 | 377.60 |
|  |  | 2 | 39.36 | 83.24 | 373.70 |
|  |  | 3 | 36.35 | 85.58 | 375.65 |
|  | -1.0 MPa | 1 | 18.75 | 58.10 | 118.80 |
|  |  | 2 | 15.96 | 60.69 | 99.00 |
|  |  | 3 | 17.35 | 59.40 | 108.90 |
|  | -1.5 MPa | 1 | 12.50 | 50.23 | 32.40 |
|  |  | 2 | 10.64 | 50.00 | 30.00 |
|  |  | 3 | 11.57 | 50.11 | 31.20 |
